# Case Presentation of a Nine-Year-Old Female With Chronic Recurrent Multifocal Osteomyelitis

**DOI:** 10.7759/cureus.38054

**Published:** 2023-04-24

**Authors:** Evanie Huang, Viktoriya G Wolfe, Susan K Yaeger, Kimberly L Fugok

**Affiliations:** 1 Department of Emergency and Hospital Medicine/University of South Florida (USF) Morsani College of Medicine, Lehigh Valley Health Network, Bethlehem, USA; 2 Department of Pediatrics/University of South Florida (USF) Morsani College of Medicine, Lehigh Valley Health Network, Bethlehem, USA; 3 Department of Emergency and Hospital Medicine/University of South Florida (USF) Morsani College of Medicine, Lehigh Valley Health Network, Allentown, USA

**Keywords:** biopsy, bone pain, diagnostic imaging, pediatrics, chronic recurrent multifocal osteomyelitis

## Abstract

Multifocal bone pain in a pediatric patient prompts a broad differential diagnosis, which should include chronic recurrent multifocal osteomyelitis (CRMO), particularly when the patient has a personal or family history of autoimmune diseases or chronic inflammatory disorders. CRMO is a difficult diagnosis, as several similar disorders must be ruled out first, and it requires extensive verification based on clinical, radiological, and pathological criteria. It often mimics other medical diagnoses, including Langerhans cell histiocytosis and infectious osteomyelitis. Maintaining a high index of suspicion for CRMO is important to minimize unnecessary medical testing, optimize pain control, and preserve physical function. We present the case of a nine-year-old female who presented with multifocal bone pain and was diagnosed with CRMO.

## Introduction

Chronic recurrent multifocal osteomyelitis (CRMO) is a rare inflammatory bone disease that primarily occurs in children and adolescents. From 1972 to 2011, more than 200 cases have been reported worldwide [1]. CRMO preferentially affects young females, with a mean age of 10 years old [1]. CRMO has been associated with autoimmune disorders of the integumentary and gastrointestinal systems, implying that there is a genetic component to its pathogenesis [2-5]. Previous studies have reported that about 50% of first- or second-degree relatives of those with CRMO have an autoimmune condition, with Crohn’s disease and psoriasis being the most common [6]. Furthermore, about 25% of CRMO patients have an associated inflammatory disorder [7].

CRMO remains a difficult diagnosis, for which similar diseases must first be ruled out via clinical, radiological, and pathological criteria, with outlined major and minor criteria [8]. The most common presenting symptom of CRMO is multifocal bone pain with or without swelling, predominantly involving the lower limbs [1]. Up to 20 known sites can be affected, including the clavicles, mandible, pelvis, and vertebral bodies [6]. Systemic symptoms, such as fever and weight loss, may be present, but objective findings on a physical exam are often absent [9]. Lab findings are largely normal, with possible mild leukocytosis and elevations in erythrocyte sedimentation rate (ESR) and tumor necrosis factor-α (TNF-α). Blood and bone cultures are also typically unremarkable.

CRMO is a radiographically diverse entity, ranging from lesions characteristic of bone lysis, sclerosis, or bone collapse [2,9]. Sclerotic lesions are most commonly found on the clavicles and mandible, while vertebral involvement is associated with bone collapse [10]. Histopathology findings range from acute, subacute, and chronic inflammation but do not correlate with clinical features [10]. A biopsy is typically the most reliable method of ruling out malignant disorders and infectious etiologies.

Treatment modalities have evolved substantially within the last two decades as more historical data regarding this rare disease has become available. Nonsteroidal anti-inflammatory agents (NSAIDS) remain the most common first-line treatment, but a combination of tumor necrosis factor inhibitors and disease-modifying anti-rheumatic drug therapies has become increasingly popular [7]. There has been a corresponding decrease in the use of surgical interventions, antibiotics, and hyperbaric oxygen to treat CRMO [7]. Available literature suggests that by 18-21 months post-therapy, a patient’s symptoms can be expected to return to baseline [10]. Without prompt and effective treatment, CRMO can cause permanent and progressively worsening disabilities among those afflicted.

## Case presentation

**Figure 1:**
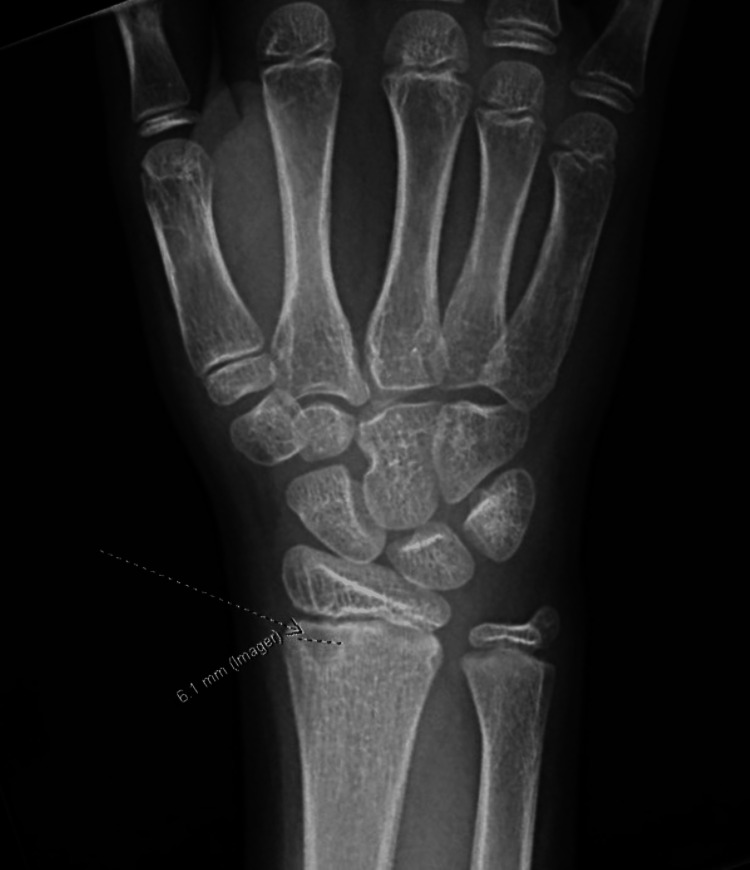
X-ray highlighting a 6.1 mm lytic lesion found on the right distal radius (arrow).

**Figure 2:**
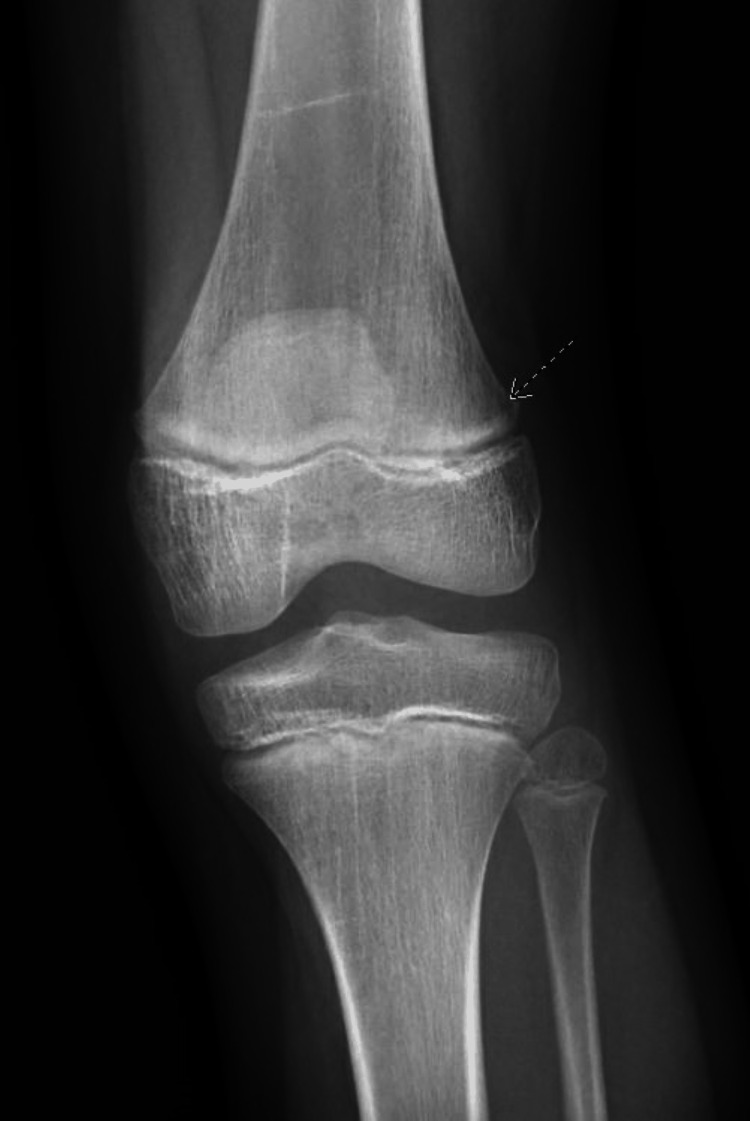
X-ray imaging showing a lytic lesion found on the distal metaphysis of the left femur (arrow).

**Figure 3:**
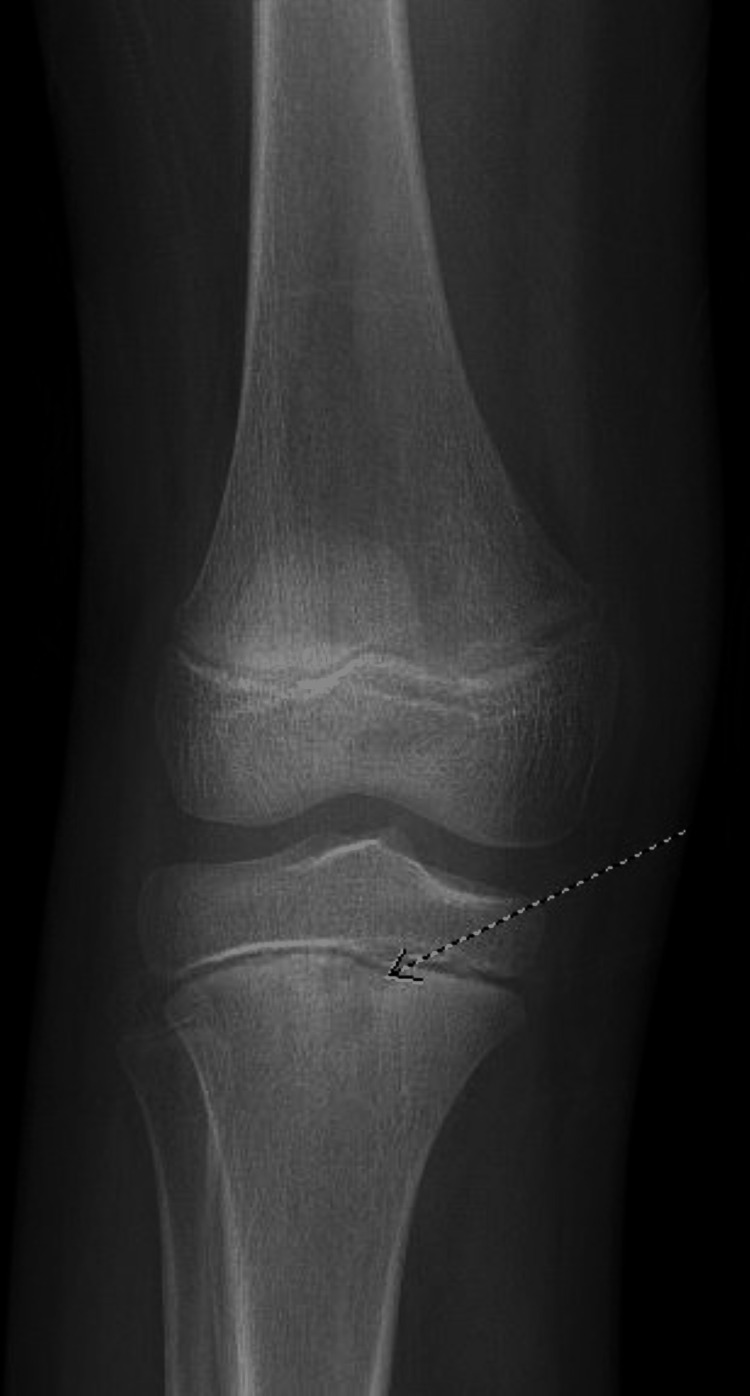
A lytic lesion (highlighted by the arrow) found on the right proximal tibia.

A previously healthy nine-year-old female with a past medical history significant for celiac disease and intermittent headaches presented to the ED with complaints of fevers, fatigue, and joint pain in her right hip, right knee, right wrist, and left ankle. Her symptoms began two weeks prior with a fever, chills, cough, diarrhea, and sore throat. Since they began immediately after her mother’s COVID infection, PCR testing was performed, which showed that the patient was COVID-negative. Her respiratory and gastrointestinal symptoms resolved within two weeks of their onset, but she continued to have intermittent fevers as high as 38.9 °C. The pain waxed and waned, causing nighttime awakenings that significantly affected her daily activities. Just prior to the ED presentation, she was prescribed a five-day course of prednisone with the suspicion of reactive arthritis after her recent gastrointestinal illness from her primary physician. Initially, pain and fever improved, only to return upon completion of the steroid course, prompting the ED evaluation. On presentation, her pain was rated as moderate, and though she still remained mobile, she was unable to ambulate normally. Acetaminophen provided only minor relief from her symptoms. There was no erythema overlying the joints and no rash, but there was mild edema and tenderness of the left ankle. The patient and parent denied recent trauma, night sweats, weight loss, emesis, diarrhea, abdominal pain, cough, congestion, rhinorrhea, sore throat, difficulty breathing, easy bruising, bleeding, or petechiae. After three weeks of symptoms, X-rays of her right wrist and both knees revealed multiple lytic lesions (Figures 1-3).

**Figure 4:**
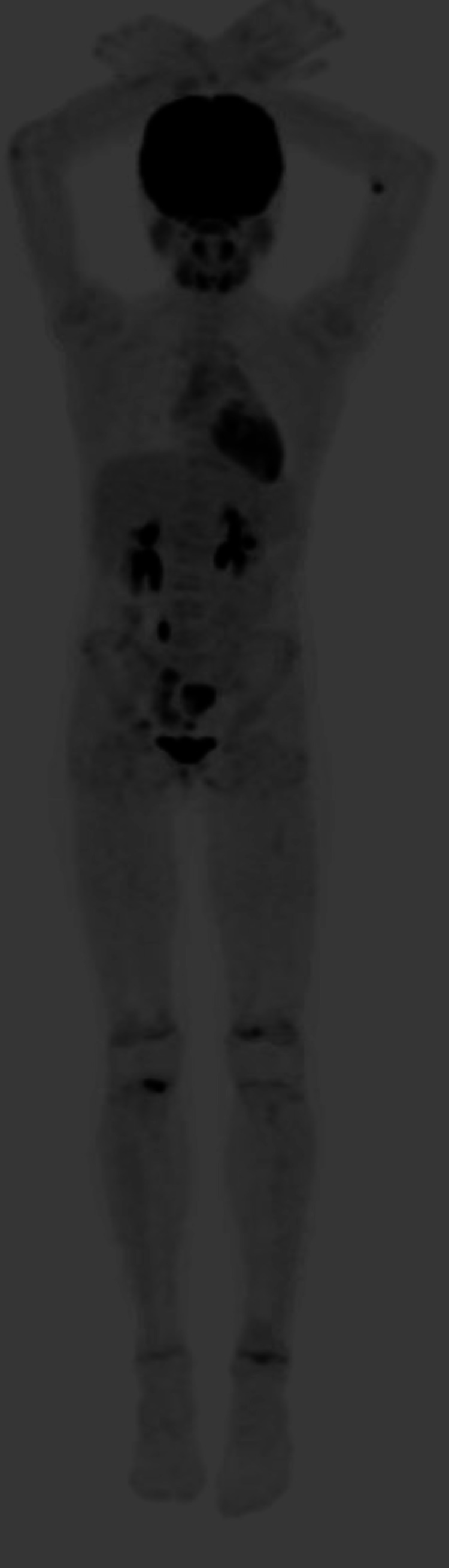
PET CT scan using F-18 fludeoxyglucose to highlight areas of increased metabolic activity. Metabolically active lytic osseous lesions can be found within the axial and appendicular skeleton.

Due to persistent fevers and her inability to ambulate as the pain progressed, she was admitted for additional workup and management. The differential diagnosis included systemic juvenile idiopathic arthritis, systemic lupus erythematosus, Ewing sarcoma, osteosarcoma, Langerhans cell histiocytosis (LCH), osteomyelitis, Brodie’s abscess, CRMO, septic arthritis, and post-strep reactive arthritis. Post-strep-reactive arthritis was included due to a positive strep test two to three months prior to presenting to the ED. Diagnostic lab work included a complete blood count, a complete metabolic panel, C-reactive protein levels, an autoimmune profile (antinuclear, Ro, La, dsDNA, SM/RNP, SCL-70, and Smith autoantibodies), mycoplasma titers, prothrombin, a prothrombin time test, an INR, and blood cultures. All of these were unremarkable. Her sedimentation rate was elevated at 34 mm/hour (reference range: 3-13 mm/hour). Immunoglobulin A was elevated at 229 (reference range 47-221 mg/dL), and immunoglobulin E was negative at <60 IU/mL (reference range <60 IU/mL). A PET scan revealed metabolically active lesions in the right proximal tibia, left distal tibia, left distal femur, right distal radius, sacrum (S5), and right acetabulum (Figure 4).

Two different bone biopsies showed foamy histiocytes and mixed inflammatory cells. Cultures performed using the biopsied bone were negative. Once tests and cultures were complete, she was diagnosed with CRMO. Her pain was controlled with NSAIDS, acetaminophen, and short-acting oral opioids. The patient was managed by pediatric rheumatology as an outpatient and started on oral steroids and bisphosphonates. The patient’s pain was difficult to control but has since improved with the addition of adalimumab. In addition to medical management, she was referred to physical therapy, which significantly improved her ambulatory function, though at six months post-diagnosis, she had still not returned to her baseline activity level.

## Discussion

CRMO is a disease that often mimics other medical conditions, including LCH, osteomyelitis, Ewing’s sarcoma, metastatic neuroblastoma, and hematolymphoid malignancy. In particular, CRMO closely resembles, both clinically and radiologically, infectious osteomyelitis and LCH. It can be a challenge to differentiate CRMO from infectious osteomyelitis based on clinical and laboratory findings, but children with bacterial osteomyelitis will frequently present with local inflammatory signs and possible abscesses [5]. Those diagnosed with CRMO were more likely to present with peripheral arthritis, inflammatory bowel disease, and hyperostosis [5,11].

LCH can occur at any age but is most commonly seen between the ages of one and four, with a slight male predominance [12]. LCH has a variable presentation, from isolated skin manifestations to multiorgan involvement. Skeletal involvement occurs in 80% of those diagnosed with LCH. LCH often involves the axial skeleton, with more than 50% of lesions occurring in flat bones such as the skull and pelvis [13,14]. The multifocal nature of her bone pain, the location of the lesions, the lack of oncologic or bacterial findings, and her past history of autoimmune disorders cumulatively led to the diagnosis of CRMO.

Once CRMO is accurately diagnosed, emphasis is placed on pain management and improving the patient’s physical function. Consultation with pediatric rheumatology, physical medicine, and rehabilitation can also help manage these patients. First-line pharmacological therapy for pain control includes NSAIDs, followed by oral corticosteroids. NSAIDS can help with both pain and the prevention of bone damage [8]. Second-line treatment includes methotrexate and may extend to biologics such as etanercept, anakinra, infliximab, adalimumab, or bisphosphonates [8]. In this case, our patient responded well to adalimumab after first-line treatments failed to adequately manage her pain and inflammation. In a pediatric patient presenting with multifocal bone pain, CRMO should remain on the differential diagnosis, as a timely diagnosis will eliminate unnecessary diagnostic procedures and medical therapies, prevent bone damage, and limit functional decline.

## Conclusions

CRMO is a rare inflammatory bone condition that must be considered in pediatric patients presenting with multifocal bone pain. Because CRMO is difficult to diagnose, it is imperative that patients with bone pain undergo a comprehensive workup to rule out other causes, such as infection, malignancy, and Langerhans cell histiocytosis. A biopsy may be considered to aid in narrowing the differential. Treatment for CRMO includes pain management, supportive care, and an early referral to appropriate specialists. The appropriate workup and timely diagnosis of patients with CRMO are essential steps in improving the patient’s quality of life and preventing joint destruction and functional loss.

## References

[1] Sadeghi E, Kadivar MR, Ghadimi moghadam AK, Pooladfar GR, Sadeghi N (2011). Chronic recurrent multifocal osteomyelitis: a case report. Iran Red Crescent Med J.

[2] Hofmann SR, Kapplusch F, Girschick HJ, Morbach H, Pablik J, Ferguson PJ, Hedrich CM (2017). Chronic recurrent multifocal osteomyelitis (CRMO): presentation, pathogenesis, and treatment. Curr Osteoporos Rep.

[3] Golla A, Jansson A, Ramser J (2002). Chronic recurrent multifocal osteomyelitis (CRMO): evidence for a susceptibility gene located on chromosome 18q21.3-18q22. Eur J Hum Genet.

[4] Jansson A, Renner ED, Ramser J (2007). Classification of non-bacterial osteitis: retrospective study of clinical, immunological and genetic aspects in 89 patients. Rheumatology (Oxford).

[5] Schnabel A, Range U, Hahn G, Siepmann T, Berner R, Hedrich CM (2016). Unexpectedly high incidences of chronic non-bacterial as compared to bacterial osteomyelitis in children. Rheumatol Int.

[6] Ferguson PJ, Sandu M (2012). Current understanding of the pathogenesis and management of chronic recurrent multifocal osteomyelitis. Curr Rheumatol Rep.

[7] Beck NA, Roudnitsky E, Nuzzi LC, Padwa BL, Dedeoglu F (2023). How have the diagnosis and treatment of chronic recurrent multifocal osteomyelitis changed over time?. J Oral Maxillofac Surg.

[8] Gicchino MF, Diplomatico M, Granato C, Capalbo D, Marzuillo P, Olivieri AN, Miraglia Del Giudice E (2018). Chronic recurrent multifocal osteomyelitis: a case report. Ital J Pediatr.

[9] Wipff J, Adamsbaum C, Kahan A, Job-Deslandre C (2011). Chronic recurrent multifocal osteomyelitis. Joint Bone Spine.

[10] Chow LT, Griffith JF, Kumta SM, Leung PC (1999). Chronic recurrent multifocal osteomyelitis: a great clinical and radiologic mimic in need of recognition by the pathologist. APMIS.

[11] Wipff J, Costantino F, Lemelle I (2015). A large national cohort of French patients with chronic recurrent multifocal osteitis. Arthritis Rheumatol.

[12] Jezierska M, Stefanowicz J, Romanowicz G, Kosiak W, Lange M (2018). Langerhans cell histiocytosis in children - a disease with many faces. Recent advances in pathogenesis, diagnostic examinations and treatment. Postepy Dermatol Alergol.

[13] Krooks J, Minkov M, Weatherall AG (2018). Langerhans cell histiocytosis in children: History, classification, pathobiology, clinical manifestations, and prognosis. J Am Acad Dermatol.

[14] Haupt R, Minkov M, Astigarraga I (2013). Langerhans cell histiocytosis (LCH): guidelines for diagnosis, clinical work-up, and treatment for patients till the age of 18 years. Pediatr Blood Cancer.

